# Nourishing future generations: the critical role of healthy school lunches and strategic food procurement

**DOI:** 10.1186/s12916-026-04708-z

**Published:** 2026-05-05

**Authors:** Aurora Perez-Cornago, Carmen Piernas

**Affiliations:** 1 European Commission, Joint Research Centre (JRC), Ispra, Italy; 2 Department of Biochemistry and Molecular Biology II, Faculty of Pharmacy, Institute of Nutrition and Food Technology, Center for Biomedical Research, University of Granada, Granada, 18016 Spain; 3 Instituto de Investigación Biosanitaria Ibs.GRANADA, Granada, Spain; 4 CIBER Physiopathology of Obesity and Nutrition (CIBEROBN), Instituto de Salud Carlos III, Madrid, Spain

**Keywords:** School meals, Public food procurement, Healthy meals, Sustainable diets, School lunches, Food policy, Meal planning

## Background

Childhood nutrition plays a crucial role in shaping the health and wellbeing of future generations. According to the latest report from the 2025 WHO European Childhood Obesity Surveillance Initiative, approximately 25% of European children aged 7–9 years were affected by overweight or obesity, and around 11% were living with obesity, based on findings from 2022 to 2024 [1]. Healthy diets, characterized by regular consumption of nutrient-dense foods such as fruits, vegetables, whole grains, and other minimally processed foods, are essential for optimal growth and development in children and for supporting the maintenance of a healthy weight. However, a WHO report revealed that fewer than half of children (46%) consume fresh fruit, and only 32% eat vegetables every day [1]. Socio-economic disparities further exacerbate these trends, as children from disadvantaged backgrounds are more likely to have limited access to healthy and nutritious foods, with potentially severe consequences for their health and development [2].

Schools, as a primary environment where children spend a substantial amount of time and consume up to a third of their total daily energy intake [3], present a unique opportunity to foster healthy eating habits and promote health. In their recent BMC Medicine publication, Dijkstra and colleagues present results from an intervention study in which Dutch primary schools introduced a free healthy school lunch, examining its effects on students’ dietary intake, their preferences, and possible compensation in eating behaviours at home [4]. The study found that offering healthy lunches in schools increases children’s healthy food intake without triggering unhealthy compensation at home, but this positive effect is reversed when the programme ends.

Here, we expand on the need for the continuous provision of healthy lunches in schools to enable children to meet nutritional targets, and we discuss the transformative potential of strategically designed food procurement contracts in promoting healthier school food environments.

## Main text

The study by Dijkstra et al. implemented a quasi-experimental pre- and post-design in 3 schools, involving 312 children aged 8–12 years and their parents. Children received a free ad libitum school lunch with healthy choices for 6 months, and photographs of the lunches and questionnaires filled by the parents were collected at baseline and 3-, 6-, and 12-month follow-ups. The study reported significant increases in the consumption of vegetables, whole grains, water, and milk following implementation, along with decreases in white bread and sweetened beverages at months 3 and 6. These changes were accompanied by high levels of student acceptability and no substantial perceived compensatory effects at home, as reported by parents. The study also showed that once the programme ended, the consumption of healthy food decreased, while the consumption of less healthy food increased [4]. This finding reinforces the need for sustained provision of healthy lunches in schools and underscores the importance of maintaining behaviour change through healthy home food environments and parental engagement.

The study by Dijkstra [4] and colleagues had several limitations, including a small number of participating schools, the absence of control schools for comparison of dietary intake, and reliance on dietary data from a single day. Nevertheless, the dietary improvements reported are consistent with findings from similar studies [5–7] and add new evidence emphasizing the need for ongoing healthy lunch programmes. In combination with targeted school food environment policies (e.g. food- and meal-based standards) [8], this study supports the notion that the direct and sustained provision of healthy food helps promote healthier diets in children. However, the longer-term benefits of these dietary improvements may also be influenced by household-level factors, including access to nutritious foods and socio-economic conditions that shape families’ ability to support healthy eating.

Public food procurement is a powerful tool for promoting healthy food options in school settings. Public food procurement refers to the process by which governments and other public institutions purchase food for schools, hospitals, and other public facilities. The integration of nutritional criteria into school food procurement processes can facilitate the provision of healthy and sustainable meals, thereby supporting the health and wellbeing of children. This can be achieved through the strategic design of public food procurement contracts that integrate technical specifications (mandatory requirements) and award criteria that incentivize the provision of healthy and sustainable food options.

To maximize the impact of such policies, it is essential that nutritional criteria are evidence based, aligned with the latest dietary recommendations, and comprehensive, with specific guidelines for implementations; however, this is currently not the case in several European countries [9]. To address this gap and promote sustainable public food procurement in the EU, the European Commission developed nutritional criteria as part of sustainable public food procurement to promote healthy and sustainable food options in European public settings [10]. Specific nutritional standards for children are outlined, such as limits on free sugars and salt. The criteria also include that fruits and vegetables shall be offered daily in half-day catering, which includes school meals, and that grains and grain products shall be offered in whole-grain form on at least half of the catering days [10].

The development of these voluntary criteria is an important step towards promoting healthy and sustainable food options in European public settings, including schools. Moreover, providing healthy school meals helps reduce inequality as it is accessible to all children, regardless of socio-economic status. Further research and analysis to assess the effectiveness of incorporating nutritional criteria into public procurement contracts on school and other public settings is encouraged. This research should focus on evaluating the impact of these and other nutritional criteria on improving school and other public sector diets, as well as identifying areas for improvement for their implementation. Moreover, it is likely that national, regional, and local authorities will require more guidance and support to implement these criteria in practice, which could be informed by the findings of this research. All this would further inform policy decisions and ultimately contribute to the successful promotion of healthy and sustainable food options in school settings.

## Conclusions

In summary, continuous provision of healthy lunch options in school settings may help achieve nutritional targets and prevent diet-related disease in future generations. Integrating nutritional criteria into school food procurement policies can play a pivotal role in shaping the dietary habits and health of future generations. Further research and evaluation are needed to ensure the success of this approach and to inform policy decisions that support the wellbeing of schoolchildren.

## Data Availability

Not applicable. No new datasets were generated or analysed for this commentary.

## References

[1] WHO European Childhood Obesity Surveillance Initiative (COSI) Report on the sixth round of data collection, 2022–2024. Regional office for Europe: World Health Organization; 2025.

[2] Gautam N, Dessie G, Rahman MM, Khanam R. Socioeconomic status and health behavior in children and adolescents: a systematic literature review. Front Public Health. 2023;11:1228632. 37915814 10.3389/fpubh.2023.1228632 PMC10616829

[3] Jaeger V, Koletzko B, Luque V, Ferré N, Gruszfeld D, Gradowska K, et al. Distribution of energy and macronutrient intakes across eating occasions in European children from 3 to 8 years of age: The EU Childhood Obesity Project Study. Eur J Nutr. 2023;62:165–74. 35930067 10.1007/s00394-022-02944-6 PMC9899743

[4] Dijkstra SC, Rongen FC, van Kleef E, Ummels M, Kuiper L, Seidell JC, et al. Improving the school food environment and dietary intake of Dutch primary school students through the provision of a healthy school lunch: results of a pre-posttest effectiveness study. BMC Med. 2025;23:1–13. 41136973 10.1186/s12916-025-04407-1 PMC12553221

[5] Andersen R, Biltoft-Jensen A, Christensen T, Andersen EW, Ege M, Thorsen AV, et al. Dietary effects of introducing school meals based on the New Nordic Diet–a randomised controlled trial in Danish children. The OPUS School Meal Study. Br J Nutr. 2014;111:1967–76. 24709026 10.1017/S0007114514000634

[6] Golley R, Pearce J, Nelson M. Children’s lunchtime food choices following the introduction of food-based standards for school meals: observations from six primary schools in Sheffield. Public Health Nutr. 2011;14:271–8. 20731886 10.1017/S1368980010002120

[7] Bartelink NHM, van Assema P, Kremers SPJ, Savelberg HHCM, Oosterhoff M, Willeboordse M, et al. One-and two-year effects of the healthy primary school of the future on children’s dietary and physical activity behaviours: a quasi-experimental study. Nutrients. 2019;11:689. 30909515 10.3390/nu11030689 PMC6470547

[8] Micha R, Karageorgou D, Bakogianni I, Trichia E, Whitsel LP, Story M, et al. Effectiveness of school food environment policies on children’s dietary behaviors: a systematic review and meta-analysis. PLoS ONE. 2018;13:e0194555. 29596440 10.1371/journal.pone.0194555 PMC5875768

[9] Garcia Herrero L, Casonato C, Perez-Cornago A, Sanye Mengual E, Sarasa Renedo A, Bakogianni I, et al. Overview and analysis of sustainable product procurement criteria in the EU food sector. Publications Office of the European Union. 2024. https://data.europa.eu/doi/10.2760/1286793.

[10] Garcia Herrero L, Perez-Cornago A, Casonato C, Sarasa Renedo A, Bakogianni I, Wollgast J, et al. Criteria for sustainable public procurement (SPP) for food, food services, and vending machines. Publications Office of the European Union, Luxembourg. 2025. https://data.europa.eu/doi/10.2760/0895877, JRC139495.

